# SEX AS A BIOLOGICAL VARIABLE IN CARDIAC AGING: CORRELATIONS AMONG ECHOCARDIOGRAM, FRAILTY, AND ANESTHESIA RECOVERY

**DOI:** 10.1093/geroni/igae098.3714

**Published:** 2024-12-31

**Authors:** Grecia Garcia Marquez, Thuy Pham, Katarzyna Cieslik, George Taffet

**Affiliations:** Houston Methodist Hospital, houston, Texas, United States; Houston Methodist Hosptital, Houston, Texas, United States; Methodist Hospital Research Institute, Houston, Texas, United States; Houston Methodist Hospital, Houston, Texas, United States

## Abstract

Cardiovascular dysfunctions are associated with advanced age. By 2050, the number of people older than 65 will triple. With this increased number of older individuals, it becomes crucial to understand how different organs respond to aging and if the processes are different between men and women. If so, then we need sex-specific approaches to extend patients’ healthspan and lifespan through effective management and treatment strategies. We and others have found sex-specific distinct cellular responses in cardiac aging in mice. In this study, we examined the correlation between anesthesia recovery time, frailty index, and cardiac parameters obtained by echocardiography and Doppler imaging performed under 1.5% isoflurane anesthesia in 25-month-old mice. Post-anesthesia recovery time and the frailty index (involving recordings of 24 different parameters) are markers of “whole body resilience”. Using matrix analysis with non-parametric Spearman correlation with two-tailed p values, we found notable differences between sexes. In males, a strong correlation was observed between diastolic parameters, anesthesia recovery time, and frailty index. In females, the correlation was strongest for systolic parameters. These findings suggest that anesthesia recovery time and frailty index are associated with different cardiac parameters in older male and female mice, underscoring the implications of cardiovascular aging differing between sexes and highlighting the importance of consideration of sex as a biological variable (SABV) in geroscience research.

